# Rewilding with invertebrates and microbes to restore ecosystems: Present trends and future directions

**DOI:** 10.1002/ece3.7597

**Published:** 2021-05-02

**Authors:** Peter Contos, Jennifer L. Wood, Nicholas P. Murphy, Heloise Gibb

**Affiliations:** ^1^ Department of Ecology Environment and Evolution, and Centre for Future Landscapes School of Life Sciences La Trobe University Melbourne Vic. Australia

**Keywords:** ecological restoration, ecosystem function, invertebrate conservation, invertebrates, microbes, revegetation, rewilding, soil inoculation, whole‐of‐community rewilding

## Abstract

1. Restoration ecology has historically focused on reconstructing communities of highly visible taxa while less visible taxa, such as invertebrates and microbes, are ignored. This is problematic as invertebrates and microbes make up the vast bulk of biodiversity and drive many key ecosystem processes, yet they are rarely actively reintroduced following restoration, potentially limiting ecosystem function and biodiversity in these areas.
2. In this review, we discuss the current (limited) incorporation of invertebrates and microbes in restoration and rewilding projects. We argue that these groups should be actively rewilded during restoration to improve biodiversity, ecosystem function outcomes, and highlight how they can be used to greater effect in the future. For example, invertebrates and microbes are easily manipulated, meaning whole communities can potentially be rewilded through habitat transplants in a practice that we refer to as “whole‐of‐community” rewilding.
3. We provide a framework for whole‐of‐community rewilding and describe empirical case studies as practical applications of this under‐researched restoration tool that land managers can use to improve restoration outcomes.
4. We hope this new perspective on whole‐of‐community restoration will promote applied research into restoration that incorporates all biota, irrespective of size, while also enabling a better understanding of fundamental ecological theory, such as colonization and competition trade‐offs. This may be a necessary consideration as invertebrates that are important in providing ecosystem services are declining globally; targeting invertebrate communities during restoration may be crucial in stemming this decline.

## Graphical abstract

Restoration and rewilding projects have historically overlooked the role played by invertebrates and microbes in restoring ecosystems. Here, we review the current state of microbial and invertebrate rewilding projects and provide recommendations on how projects can move forward in the future.

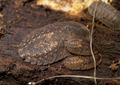

## 1 INTRODUCTION

Globally, ecosystems have suffered extensive, largely negative change through human activity. In efforts to ameliorate our impact, we invest billions into ecological restoration each year to repair environments (Palmer et al., 2016). Although there has been considerable discussion concerning the goals of such large monetary investments (including debate around embracing novel communities or aiming for a predisturbance remnant site (Hobbs et al., 2009), see Section 5), there are clear trends in how we have approached restoration so far. For example, although ecological restoration is the process of whole‐ecosystem recovery, plant‐only restoration dominates current practices (67% of projects) with only 24% of projects restoring both plants and animals simultaneously (McAlpine et al., 2016) (9% of projects were animal‐only restoration and this likely occurs when the plant community is already in good condition). This focus on plants suggests that ecosystems are expected to conform with the “Field of Dreams” paradigm that is embedded within restoration ecology (Palmer et al., 1997; Prach et al., 2019), that is, if you build the habitat, other organisms will recolonize passively.

Plants also receive considerably more attention than nonplants in postrestoration monitoring: Plants are surveyed in 54% of projects, whereas less visible groups such as invertebrates and microbes are monitored in only 32% of projects (27% and 5%, respectively) (Kollmann et al., 2016). Studies of passive recolonization suggest that, although many species do recolonize without additional effort (Barber et al., 2017; Wodika et al., 2014), there are many factors that restrict fauna passively recolonizing restoration sites, most notably the suitability of the restored habitat, the proximity to source populations, and dispersal limitations of some fauna (Kitto et al., 2015; Parkyn & Smith, 2011). Dispersal limitations may be especially pertinent in reconstructing communities postdisturbance for smaller organisms such as invertebrates and microbes which are often dispersal‐constrained (Brederveld et al., 2011; Chen et al., 2020; Jourdan et al., 2019; Peay et al., 2010).

Invertebrates and microbes are immensely important for restoration processes as they are key drivers of landscape‐scale ecosystem functions such as nutrient cycling (Eisenhauer, 2019) and carbon sequestration (Anthony et al., 2020). However, it is often assumed that they colonize independently following restoration of plant species (Strickland et al., 2017). Although some invertebrates and microbes passively recolonize revegetated areas (Barber et al., 2017; Wodika et al., 2014), not all species can disperse to, colonize, or establish successfully. Indeed, invertebrate and microbe communities in revegetated areas do not often become indistinguishable from those in remnant sites. Some macroinvertebrate communities in restoration sites are only ~35% similar to reference sites 20 years postrestoration, whereas the relative abundance of key bacterial Phyla was only half recovered as compared to nearby target sites 16 years postrestoration (Strickland et al., 2017; Wodika & Baer, 2015). Although some of this difference is likely related to the complex interaction between temporal changes in habitat suitability and the movement of metacommunities, a significant proportion may be due to dispersal limitations (Kitto et al., 2015). For example, dispersal constraints have been suggested as a limiting factor in recolonization of restored streams by macroinvertebrates (Brederveld et al., 2011), restored meadows by snails (Knop et al., 2011), and restored arable land by microbes (Chen et al., 2020).

Where passive recolonization fails, more proactive attempts to improve ecosystem function and biodiversity in revegetated areas include actively reintroducing or “rewilding” missing biota. Rewilding is an increasingly popular conservation tool whereby select fauna are reintroduced to reinstate ecosystem function and restore degraded areas (Corlett, 2016). Although a relatively new term, the concept of rewilding can be seen as a subset of restoration (Hayward et al., 2019). As such, rewilding is similarly biased toward highly visible groups (vertebrates in this instance), with comparatively few published examples of rewilding with less obvious groups such as invertebrates and microbes. In the related field of reintroduction biology, invertebrates make up as little as 3% of reintroduction studies, despite their roughly 95% contribution to species diversity (Bajomi et al., 2010). Rewilding projects have therefore tended to ignore the “unseen majority”: functionally important yet overlooked groups such as invertebrates and microbes. Examples of invertebrate and microbial rewilding are however common in soil inoculation studies, which often rewild whole communities during soil transplants. There are significant knowledge gaps within these studies as few monitor changes in invertebrates and microbes postsoil inoculation. The effect of rewilding was thus difficult to quantify in these instances (see Section 3). The potential for rewilding dispersal‐constrained invertebrates and microbes into areas they fail to recolonize naturally is under‐researched outside of soil inoculation studies and is therefore rarely considered during restoration. However, rewilding may increase the likelihood of achieving restoration goals, particularly where the aim is to restore to a state of biodiversity and ecosystem function that is similar to the source area.

## 2 OBJECTIVES

In this review, we discuss the current incorporation of invertebrates and microbes into rewilding and restoration projects and how their use can be improved in the future. First, we explore how invertebrates and microbes have been used in ecosystem restoration to date and provide a summary table that highlights significant knowledge gaps in our approach to invertebrate and microbial rewilding so far. Next, as rewilding has significant ecosystem ramifications (both intentional and unintentional), we discuss scenarios in which invertebrate and microbial rewilding is justified during restoration. Finally, we discuss how invertebrate and microbial rewilding can move forward in the future by utilizing their unique characteristics. This includes specific examples of empirical invertebrate and microbial rewilding projects that land managers can use during restoration to improve the recovery of ecosystem functions and biodiversity. Our goal is to challenge the current plant‐focused view of restoration and provide the foundations for a more holistic approach that better values the role of invertebrates and microbes during ecosystem recovery.

## 3 ACTIVE RESTORATION OF INVERTEBRATES

The return of invertebrates to revegetated areas is crucial for restoration goals as they are critical components of functioning ecosystems. Invertebrates may fail to actively recolonize due to inadequate habitat within the restoration site or characteristics that limit dispersal, such as a lack of wings (Haase & Pilotto, 2019). Regardless of the cause, proactive solutions are rarely implemented when monitoring reveals that important trophic groups have failed to recolonize revegetated sites. As such, there are few examples of active rewilding of invertebrates. The limited examples center on rewilding earthworms (usually a single species) into degraded areas to improve decomposition rates (Snyder & Hendrix, 2008). Jouquet et al. (2014) reviewed the role of earthworms (and termites) in restoration so far and highlighted their limited use (only three field studies from 1999 to 2014) and how projects could be expanded, for example, using earthworms to reduce erosion. Further, although the practice of rewilding to improve ecosystem function and biodiversity may be informed by the much larger literature on invertebrate translocations, the intention of this practice is very different. Species translocations are usually conducted for species conservation and the functional role of the species is rarely considered, let alone assessed (Bellis et al., 2019). Invertebrates targeted for translocations are often large, charismatic endangered species (such as Wetas and butterflies), with smaller, functionally important, taxa ignored. Similarly, there are emerging studies noting the effect of trophic rewilding on invertebrates and microbes which differ from the points raised in this review (Gibb et al., 2021; van Klink & WallisDeVries, 2018; Andriuzzi & Wall, 2018). These studies examine the effect of rewilding other biota on invertebrate and microbe communities, rather than directly manipulating invertebrates and microbes via rewilding.

Entire communities of invertebrates have been reintroduced in multiple studies, although the practice is in its infancy. Topsoil inoculum contains whole communities of invertebrates (and microbes), potentially offering an avenue for community restoration. Several studies test the impacts of inoculating restoration sites with soil taken from target areas (Brown & Bedford, 1997; Lance et al., 2019; Wubs et al., 2016). Although the focus is often on changes in ecosystem function, the process of soil transplantation is in effect rewilding the whole soil invertebrate community. Yet, quantification of soil invertebrate responses to these treatments is rare, with only 29% of studies monitoring post‐transplant changes in invertebrate communities (Table 1). Those studies that have quantified invertebrate responses have shown that transplants of whole soil communities can improve the biodiversity and density of mites and springtails (van der Bij et al., 2018; Wubs et al., 2016), soil nematode abundance (Benetková et al., 2020), and soil macrofauna abundance (Moradi et al., 2018).

**TABLE 1:** We found 21 published examples where whole communities of invertebrates and microbes were reintroduced during restoration projects (ignoring mesocosm and glasshouse experiments)

Study	Target taxa	Rewilding practice	Amount of habitat used	Source of taxa	Changes in invertebrate/microbe biodiversity	Changes in function
Wubs et al. (2016)	Soil/plant communities	Soil inoculation	1–2.5 L/m^2^ (over 5,000 m^2^)	Nearby remnant	↑ richness acari/collembola, ↑ biomass microbes	↑ succession rate of desired plant species
Emam, (2016)	Soil/plant communities	Soil inoculation	0.16 L/m^2^ (over 10m^2^)	40‐year‐old stockpiled soil	Not measured	↑ soil N content
Lance et al. (2019)	Soil/plant communities	Soil inoculation	50 g/plant	Nearby remnant	Not measured	↑ soil C:N ratio and phosphorous
Lance et al. (2020)	Soil/plant communities	Soil inoculation	50 g/plant	Nearby remnant	∆ microbe community composition	Not measured
Rowe et al. (2009)	Soil/plant communities	Soil inoculation	0.16 L/m^2^ (over 1.5 m^2^)	Nearby remnant	Not measured	∆ plant community composition
Soteras et al. (2014)	Soil/plant communities	Soil inoculation	30 g/plant	Nearby remnant	No microbe community changes detected	No changes in primary productivity
Grove et al. (2019)	Soil/plant communities	Soil inoculation	3 L/plant	Nearby remnant	No microbe community changes detected	No changes in above ground biomass
van der Bij et al. (2018)	Soil/plant communities	Sod inoculation	3.33 L/m^2^ (over 15 m^2^)	Nearby remnant	↑ acari, nematode, collembola abundance/∆ microbe community	Not measured
Benetková et al. (2020)	Soil/litter communities	Soil and litter inoculation	33% surface coverage of 100 m^2^ plots	Nearby remnant	↑ number of nematode genera	Not measured
Moradi et al. (2018)	Soil communities	Soil inoculation	400 L/m^2^ (over 30 m^2^)	Nearby remnant	↑ number of earthworms and millipedes	↓ soil carbon and C:N ratio than overburden soil
Faist et al. (2020)	Biocrust communities	Soil inoculation	0.5 L/m^2^ (over 3 m^2^)	Nearby remnant	Not measured	Weak ↑ in soil stability, highly spatially dependent
Chiquoine et al. (2016)	Biocrust communities	Soil inoculation	30% surface coverage of 1 m^2^ plots	Predisturbance community	↑ cyanobacteria density	Partial recovery of soil stability
Fisseha et al. (2019)	Soil communities	Rhizosphere trap cultures	Not measured	Remnant trees	Not measured	No changes in primary productivity
Pywell et al. (2011)	Plant communities	Soil inoculation	24 kg/m^2^ (over 500 m^2^)	Nearby remnant	Not measured	No significant differences in plant reassembly
Middleton and Bever, (2012)	Plant communities	Soil inoculation	13.5 ml/seedling	Nearby remnant	Not measured	↑ growth of late‐successional plants
Nishihiro et al. (2006)	Wetland plant communities	Lake sediment inoculation	100 L/m^2^ (over 5,300–27,800 m^2^)	Nearby fishing lake	Not measured (methodological paper)	Not measured
Brown and Bedford, (1997)	Wetland plant communities	Wetland soil inoculation	150 L/m^2^ (over 0.75m^2^)	Nearby remnant	Not measured	↑ plant growth
Brown et al. (1997)	Wetland macroinvertebrates	Wetland soil inoculation	100 L/m^2^ (over 550–950 m^2^)	Nearby remnant	↑ macroinvertebrate abundance	Not measured
Dumeier et al. (2020)	Freshwater benthic invertebrates	Capturing whole communities	0.05 kg/m^2^ of habitat substrate (over 500 m^2^)	Nearby remnant	Not measured (methodological paper)	Not measured
Haase and Pilotto, (2019)	Freshwater benthic invertebrates	Capturing whole communities	31,250 cm^2^ habitat per stream	Nearby remnant	Not measured (methodological paper)	Not measured
Haskell et al. (2012)	Plant communities	Dead wood transplants	50% surface coverage of 9 m^2^ plots	Nearby Remnant	Not measured	↑ plant growth/regulated soil temperatures

Note

This is excluding single species reintroductions of earthworms and termites (which are reviewed in Jouquet et al. (2014)), single species reintroduction of Arbuscular Mycorrhizal Fungi (which are reviewed in Asmelash et al. (2016)), and single species reintroductions of cyanobacteria (which are reviewed in Rossi et al. (2017)).

The paucity of invertebrate rewilding projects demonstrates that there are significant knowledge gaps regarding if, how, and when invertebrates should be used to restore ecosystem function. However, the diversity of ecosystem functions provisioned by invertebrates may be matched by an equally diverse range of situations which call for active rewilding efforts.

## 4 MICROBIAL RESTORATION: MOVING BEYOND INTERACTIONS WITH PLANTS

Like invertebrates, it is generally assumed that microbial diversity and function in revegetated areas will naturally attain the level maintained in remnant sites. However, communities of microbes are monitored the least of any organism group postrestoration (5% of projects) (Kollmann et al., 2016). For microbes, targeted reintroductions aimed at improving plant health are the focus: Inoculation of single species of non‐native Arbuscular Mycorrhizal Fungi (AMF) is a common restoration practice (Asmelash et al., 2016). Non‐native AMF are used to help revegetated plants establish and grow, but this practice ignores all other components of soil biota. Indeed, it has been argued that AMF inoculations could be improved by incorporating whole native communities rather than using single non‐native species (Asmelash et al., 2016), and this hypothesis is now being tested through field trials (Lance et al., 2019). There are analogous developments in fields that use cyanobacteria to improve soil processes. Emerging studies examining the efficacy of whole community transfer of cyanobacteria (Chiquoine et al., 2016) are challenging the traditional use of single‐strains of laboratory‐reared species (Rossi et al., 2017).

**FIGURE 1:**
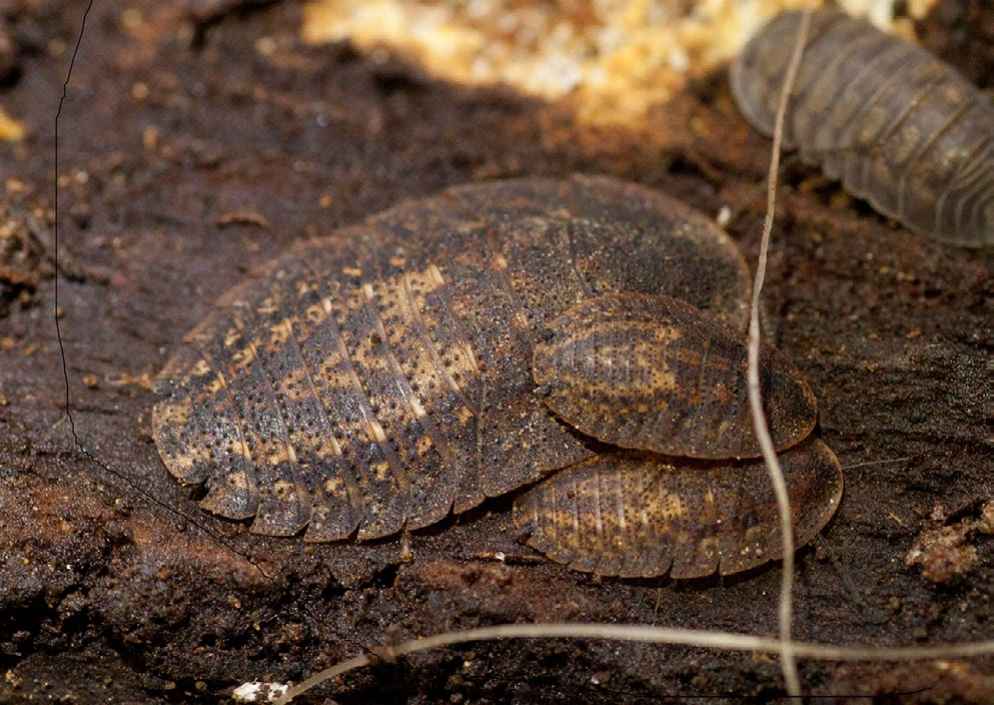
Litter communities contain a breadth of species, including trilobite cockroaches, *Laxta granicollis* (center), and armadillid isopods (top right). These taxa are often overlooked during rewilding projects, despite their immense contribution to biodiversity and their influence on ecosystem functions such as decomposition. Photo credit: L Menz

The implementation of both invertebrate and microbial rewilding projects is impeded by knowledge gaps. Addressing these gaps would include greater monitoring both postrestoration to identify which groups are failing to recolonize revegetated areas and postrewilding to determine which groups have established (Table 1). Further, for ecological restoration, it might make more sense to consider whole communities: the ultimate success for restoration would be to reinstate biodiversity and ecosystem function in its entirety. To do this, microbial rewilding will need to venture from the plant‐focused singular AMF inoculation studies, while invertebrate rewilding should broaden from earthworms to communities that include a greater diversity of functional groups, such as those found in litter (Figure 1).

## 5 WHEN IS REWILDING INVERTEBRATES AND MICROBES NECESSARY?

**FIGURE 2:**
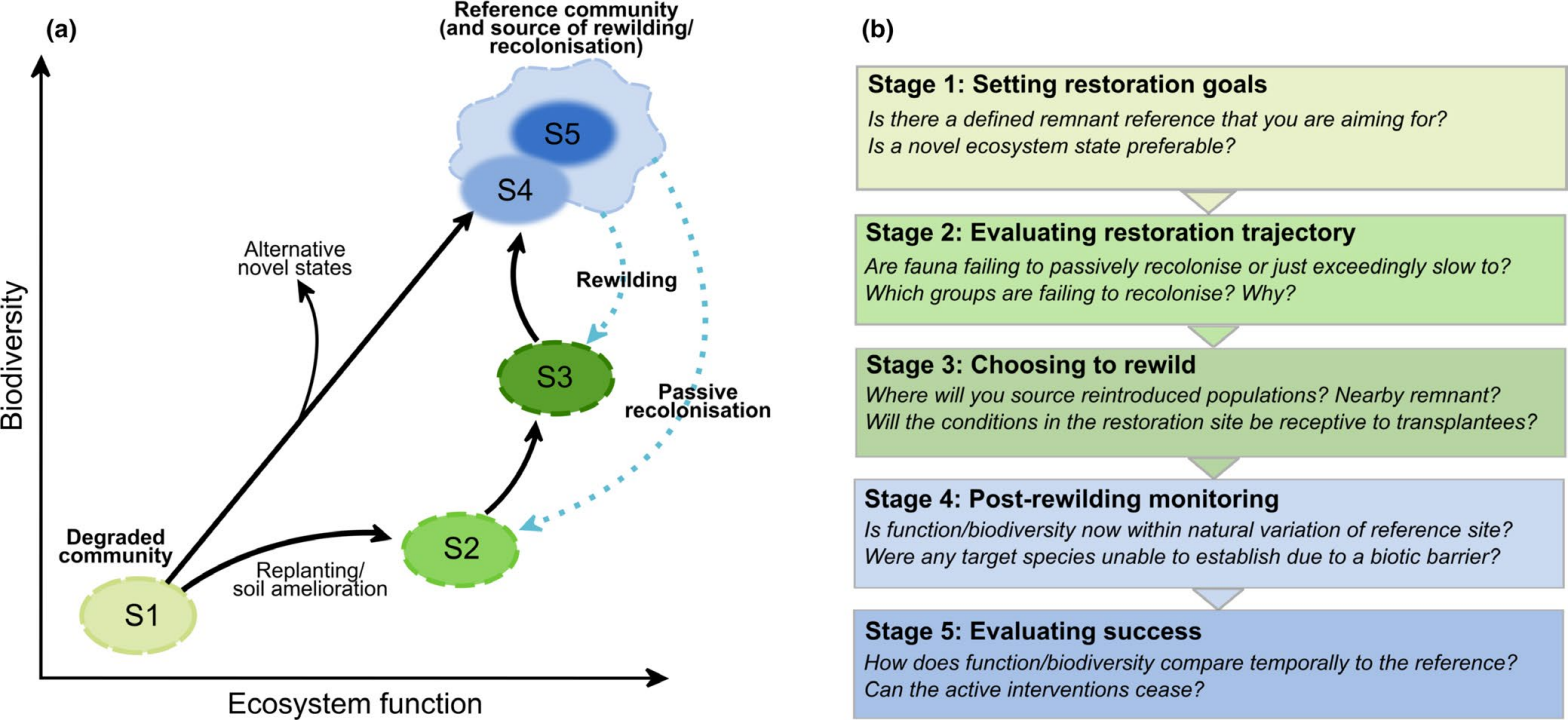
Conceptual framework of trajectories and restoration options for degraded communities modified from Bradshaw (1996) and Hobbs and Norton (1996) (a). Each step of restoration is associated with key questions practitioners need to answer to justify active interventions or to evaluate restoration goals (b). Following these stages, the degraded community (S1) is replanted with vegetation (S2). Fauna from the reference remnant community (S5) then passively recolonize the new restoration habitat. Where biodiversity and function are exceedingly slow or unlikely to reach remnant levels, active intervention via rewilding (S3) may push the restoration community closer to the reference community. Over time, biodiversity and function in the restoration community may sit within the natural variation (wavy lines) of the target reference community (S4)

Whether or not a practitioner chooses to rewild invertebrates or microbes is highly dependent on the first critical step in restoration: setting goals and targets (Prach et al., 2019) (Figure 2). For example, practitioners that accept a novel ecosystem may let a postdisturbance community form from whichever biota are best adapted to the novel abiotic conditions, regardless of their status as native to the area or their functional role, thereby avoiding active intervention (Hobbs et al., 2009). Other approaches aim to restore an area to a “natural” predefined target state in terms of species composition or ecosystem function. This is a common goal in ecological restoration and is the first of six key concepts underpinning best practice in ecological restoration as defined by the international Society for Ecological Restoration (Mcdonald et al., 2016). These target states are often based on the species, trait, and/or functional diversity of one or more nearby remnant sites, or if no remnant sites exist, literature that describes the community predisturbance (Prach et al., 2019). This paradigm is inherently interventionist as it can take significant effort and resources to push a degraded ecosystem toward its predisturbance state. As such, practitioners may be more inclined to rewild fauna from remnant sites when there is a desired remnant target state (Figure 2).

Restoration success or failure can often depend on the ability of dispersal‐limited species to reach and recolonize restoration sites and how this factor interacts with temporal changes in habitat conditions (Baur, 2014). The amelioration of microclimatic and biotic conditions over time will no doubt influence the colonization rate of restored areas. However, empirical tests of metacommunity theory demonstrate that dispersal constraints can often outweigh the importance of environmental conditions for invertebrate community structuring postrestoration. For example, Kitto et al. (2015) used metacommunity analysis to evaluate the importance of dispersal constraints versus amelioration of environmental conditions for the restoration of benthic invertebrate communities in restored streams. They found that although some environmental variables structured communities, this was independent of the effect of stream location across a landscape and the proximity to remnant source populations. Chen et al. (2020) found analogous relationships in soil microbial restoration, noting that dispersal limitation was a stronger determinant than environmental filtering for the reconstruction of archaeal, bacterial, and fungal communities postdisturbance. This demonstrates that, where restoration sites are geographically isolated from remnant sites, or where target fauna are dispersal‐constrained, rewilding can play an important role in achieving restoration goals. It is also crucially important that abiotic and biotic conditions of restoration sites are monitored prerewilding. This is not only to establish that abiotic conditions will be receptive to transplantees, but to confirm the restoration site has reduced efficiencies of an ecosystem function and/or biologically depauperate communities, thus justifying active rewilding efforts. This can be extended beyond simple monitoring methods. For example, Thierry and Rogers (2020) proposed a conceptual framework that identified priority rewilding sites based on habitat suitability, areas with inefficient ecosystem functions, and societal factors.

Given a practitioner has chosen to rewild, the next critical question is: When is it appropriate to intervene? One of the main advantages of choosing a desired target community endpoint is that you can track the trajectory of postdisturbance communities toward that of the target. The difficulty with this approach is that realistic timelines need to be set as to when these targets should be met. Some restored wetlands are over 50 years old and have only recovered 53% of their biogeochemical function as compared to remnant states (Moreno‐Mateos et al., 2012). Ecosystem recovery can take much longer than 50 years (100s–1000s years); however, restoration projects are often under pressure to demonstrate success through attainment of predefined goals (Wortley et al., 2013). Whether or not a project is failing, or just exceedingly slow to reach its goals, is a key question for restoration ecologists. Addressing this question, Parkyn and Smith (2011) hypothesized when intervention is needed and how this interacts with the dispersal capabilities of target fauna. They estimated that well‐connected restored streams in New Zealand would often reach their desired invertebrate community reference state in between 10 and 50 years, whereas poorly connected streams may never reach this state, regardless of improving environmental conditions. The latter scenario might be common in highly disturbed systems and may have stimulated emerging studies that examine the feasibility of rewilding whole communities of invertebrates into streams undergoing restoration (Dumeier et al., 2020; Haase & Pilotto, 2019).

## 6 FUTURE POSSIBILITIES FOR INVERTEBRATE AND MICROBIAL REWILDING

Stepwise restoration of communities by adding individual species is becoming increasingly unattainable, unrealistic, and ineffective in our rapidly changing and dynamic world. This has no doubt influenced the trajectory of restoration and rewilding projects, which have increasingly focused on reinstating ecosystem function and self‐organizing communities, rather than compiling set groups of species (Harris, 2014; Perino et al., 2019). This changing paradigm suits the unique characteristics of invertebrates and microbes, further encouraging their use in future rewilding projects. For example, the astounding diversity of invertebrates and microbes, the lack of knowledge of their functional roles, and their high spatial turn‐over rates means that in any given community we are often unsure of which species are functionally critical (Setälä et al., 2005). Thus, targeted rewilding of single species may not lead to desired changes in ecosystem function efficiency. However, invertebrates and microbes are miniscule and thus easily manipulated, meaning we can readily move whole communities, and the functions they provision, from one place to another (given the habitat is appropriate and enough species establish). This is already how a majority of invertebrate and microbial rewilding projects operate. For instance, soil inoculation is a common form of invertebrate and microbial rewilding which consists of moving soil from target sites (with invertebrate and microbe communities in situ) into restoration sites (Wubs et al., 2016). We term this practice “whole‐of‐community” rewilding, and although it is evident within soil inoculation studies, it is highly under‐researched outside of soil transplants and thus rarely considered during restoration (Table 1). Whole‐of‐community rewilding consists of transporting small subsets of whole habitats, complete with invertebrate and microbe communities, from desired sites into restoration areas. The desired sites are at the practitioner's discretion; thus, they can tailor the constructed community based on whichever site they choose. However, a summary of whole‐of‐community rewilding for restoration purposes highlights that nearby remnant sites are most frequently chosen (86% of projects), which conforms to mainstream restoration paradigms (i.e., choosing a nearby “intact” site as the desired endpoint community) (Table 1) (Mcdonald et al., 2016).

Even within the limited examples of whole‐of‐community rewilding, there are clear knowledge gaps and missed opportunities. For example, very few studies monitor invertebrate and microbe communities postinoculation (Table 1), making it difficult to quantify the efficacy of whole‐of‐community rewilding and its effect on community dynamics. Postreintroduction changes were only recorded in 24% of transplant projects for invertebrates and 29% of projects for microbes, highlighting that monitoring postreintroduction is crucial for greater understanding of the successes and failures of this holistic form of rewilding. Changes in ecosystem function postreintroduction were recorded more frequently (67% of projects), but it is difficult to link the effect of rewilded invertebrates and microbes to changes in function when they are not monitored. Further, invertebrates and microbes are ubiquitous, meaning there may be many more instances outside of those documented (Table 1) where whole‐of‐community rewilding may be applied. For example, litter communities are critical for efficient nutrient cycling and can be easily transported within their habitat (Silva et al., 2020). However, litter transplants with the purpose of improving decomposition rates during restoration have not been attempted before (Box 1).

Although the direct mechanisms by which whole‐of‐community rewilding improves ecosystem function is likely highly contextual, this practice can influence a broad range of functions, including; nutrient dynamics (Lance et al., 2019), plant growth (Emam, 2016), and community trajectories (Wubs et al., 2016). One possible link between ecosystem function and this rewilding practice is the associated rapid increase in biodiversity. This relationship is known as the Biodiversity–Ecosystem Function (BEF) hypothesis and posits that increases in biological diversity (number of species, genotype varieties, etc.) will see similar increases in the efficiency of ecosystem functions (Srivastava & Vellend, 2005). Although debate surrounds the generality of patterns between biodiversity and ecosystem function (e.g., how the effect varies over spatial scales (Thompson et al., 2018)), it may be of particular use to restorationists as postdisturbance communities are biologically depauperate and their diversity can be easily and directly manipulated through practices such as rewilding (Srivastava & Vellend, 2005).

BOX 1 **Rewilding litter invertebrates and microbes to improve nutrient cycling**

Litter‐dwelling detritivore invertebrates and microbes are critical; yet overlooked, components of ecosystems (Bender et al., 2016; Eisenhauer, 2019). They support the breakdown of leaf litter, which turns organically bound nutrients into nutrients available for uptake by plants. Their full return to revegetated areas is therefore of paramount importance for revegetated plant communities and restoration. Litter invertebrate and microbe communities in revegetated areas sometimes track toward remnant communities (Waterhouse et al., 2014; Wodika et al., 2014), but this is not always the case (Strickland et al., 2017; Wodika & Baer, 2015) as species can have limited dispersal abilities (Peay et al., 2010). Where they fail to recolonize following restoration, active rewilding may not only improve biodiversity but also the efficiency of litter breakdown and nutrient cycling.

Increasing revegetation of farmland opens many opportunities in which dispersal constraints of litter invertebrates and microbes may justify rewilding (Gibb et al., 2012). Revegetated areas often exist as “habitat islands” surrounded by intensively managed pastures or crops. Dispersal‐constrained invertebrates and microbes can struggle to recolonize these habitat islands due to unfavorable microclimatic and biotic conditions of pastures (Pompermaier et al., 2020; Strickland et al., 2017). Revegetated areas may therefore never become indistinguishable from remnants in terms of species composition, which is critical in driving ecosystem functions such as decomposition (Schuldt et al., 2018). Active translocations of litter communities from remnant sites into revegetated areas may boost leaf litter breakdown and nutrient cycling by increasing species diversity or introducing dispersal‐limited species that are driving community differences.

**FIGURE 3:**
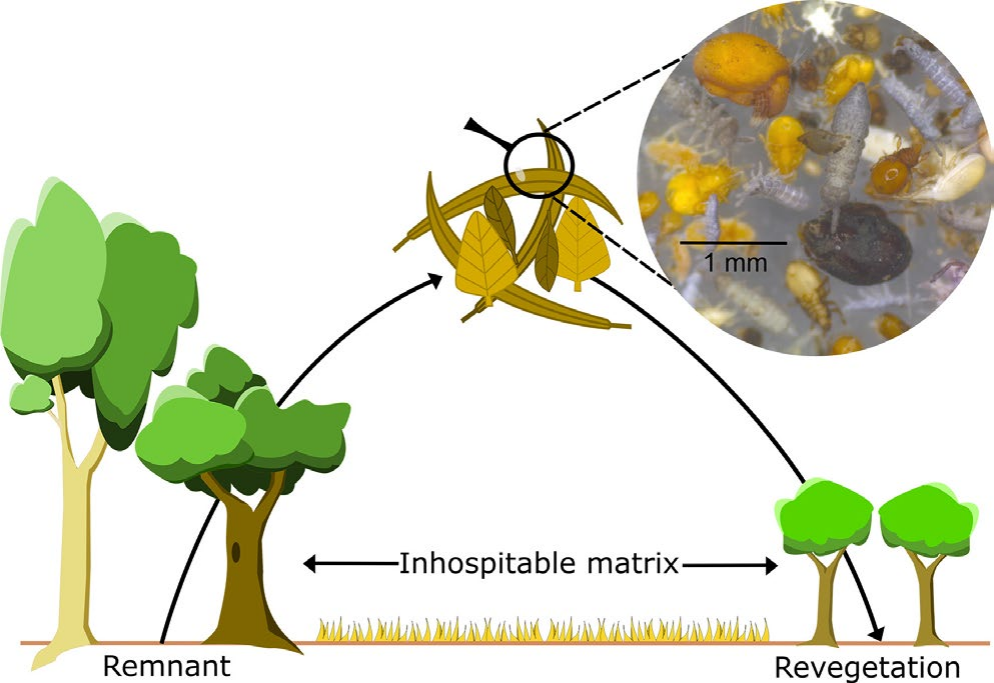
Leaf litter samples taken from remnant patches and moved into revegetation patches will carry a multitude of invertebrate and microbe species and individuals. Inset: detritivorous mites and springtails taken from a leaf litter sample

Species interaction networks in litter communities are notoriously complex. It is therefore difficult to identify keystone drivers of nutrient cycling and litter breakdown. Indeed, efficient breakdown of litter at one stage is often dependent on functions performed by different taxa at previous stages (e.g., microbial conditioning makes leaf litter more palatable for invertebrates) (Peralta‐Maraver et al., 2019). We have limited understanding of the specifics of these inter‐dependencies. Whole‐of‐community reintroductions may therefore be more appropriate to improve ecological function in revegetated areas and would entail transporting leaf litter habitat with its complete biota from remnant sites into revegetated areas (Figure 3). Timing and source of litter transplants is likely to be crucial as ecosystem functions vary spatiotemporally. For instance, litter mass loss and functional diversity of detritivores is reduced during drought conditions as litter invertebrates may enter diapause and move deeper into the litter layer, where they are less likely to be captured (Silva et al., 2020). Litter transplants will therefore be more effective at the height of detritivore activity which is generally during cool and wet conditions.

## 7 HOW CAN WE REWILD WHOLE COMMUNITIES?

Successful whole‐of‐community rewilding, and indeed any form of reintroduction, depends on the suitability of habitat to which the transplantees are moved. For whole‐of‐community rewilding, the transplants of whole habitat would ideally come from nearby remnant areas of similar original state as they are most likely to contain species appropriate to the environment of the revegetated area (Dumeier et al., 2020; Jourdan et al., 2019; Wubs et al., 2016). This both increases the likelihood of successful establishment and ensures that communities with appropriate functional and life history traits are used during restoration. Using nearby remnant target sites is the more common method for setting restoration goals (Mcdonald et al., 2016) and is how most documented cases of whole‐of‐community rewilding choose their source of rewilded populations (86%) (Table 1). Further, it is vital that environmental conditions of the restoration site have been appropriately ameliorated and are receptive to transplantees. For example, Haase and Pilotto (2019) assessed and required 21 abiotic variables of restored streams (physiochemical variables, hydromorphology, and land use) to be within specific thresholds of that of intact streams before choosing to rewild entire benthic communities of invertebrates.

Successful establishment also hinges on transferring communities at appropriate times and in appropriate quantities. This will undoubtedly vary according to the target communities. For example, when transferring whole communities of stream invertebrates, Haase and Pilotto (2019) suggest using a modified version of sampling (German EU Water Framework Directive) that adequately samples all microhabitats in a stream and to repeat this every second month for a year to capture all life stages. Similar protocols would need to be developed when transporting other elements of the community. Once transferred, successful establishment of communities depends on translocating a minimum viable population (MVP) of each species that can overcome mortality rates and inbreeding depressions. This would ideally be factored into pretranslocation thinking, with targeted monitoring of source populations to determine the amount of habitat that contains enough species and individuals to establish a new community. Although there have been attempts to develop a general MVP regardless of taxon (Flather et al., 2011), the amount of habitat collected to obtain an MVP will undoubtedly vary according to taxon, the environment from which they are sampled, and the size of the area into which they are transplanted. For example, there is great variation in the amount of transplanted habitat needed to achieve successful whole‐of‐community rewilding, which can range from 0.16 L/m^2^ (Emam, 2016) to 2.5 L/m^2^ (Wubs et al., 2016) of soil (Table 1). There would be a considerable amount of effort needed to move larger amounts of habitat, yet smaller amounts could be preferred given that transplantees successfully spread throughout the restoration site. Dispersal of transplanted individuals is highly dependent on the surrounding environmental conditions. For example, Moradi et al. (2018) found that although soil transplants lead to greater abundances of soil macrofauna, areas adjacent to transplant sites were devoid of soil macrofauna due to physical and chemical limitations of the surrounding soil.

There are clear advantages that invertebrate and microbial rewilding has over traditional (vertebrate‐focused) projects. Because invertebrates and microbes are miniscule, whole communities can be easily transported within small transplants of habitat containing a multitude of species, individuals, and propagules—bypassing the slow species‐by‐species reintroductions seen in current restoration practices (Corlett, 2016). This is inherently advantageous as the purpose of restoration is the complete return of biota and function, not just some specific species. Unlike traditional rewilding projects, whole‐of‐community rewilding also does not discriminate based on “likeability” of a species, meaning that overlooked, yet functionally important species, can be incorporated into restoration more frequently (Jourdan et al., 2019). These benefits may be why the whole‐of‐community reintroduction paradigm is ingrained in soil restoration and starting to gain traction in stream restoration (Dumeier et al., 2020; Haase & Pilotto, 2019). This is not to say that single species rewilding has no place in future projects. For instance, single species reintroductions of butterflies and bumblebees in the United Kingdom were the most feasible way to reconstruct historic pollinator communities and improve pollination across the landscape (Steele et al., 2019).

## 8 RISKS AND CONSIDERATIONS FOR WHOLE‐OF‐COMMUNITY REWILDING

There are, however, risks associated with whole‐of‐community rewilding that need to be acknowledged and addressed. Land managers should be aware of the potentially detrimental effects on remnant sites that this form of rewilding may have if it were unregulated. For example, the repeated removal of habitat from remnant areas during the rewilding event might diminish source populations of invertebrates and microbes or degrade habitats. This is especially true if the microclimatic and biotic conditions of the new restoration site are unsuitable for the transplantees as they will likely perish, meaning the only outcome of the transplant is a diminishment in source populations as opposed to an increase in their range. This could be remedied by regulation of the harvesting in space and time, for example, by spreading the collection event over various areas within the remnant sites, ensuring that (a) source populations are not depleted; (b) transplanted populations capture the breadth of diversity and life history stages in natural communities; and (c) interacting co‐dependent taxa are introduced together.

Land managers will also need to be conscious of the potential to spread invasive invertebrates and microbes during the rewilding event as non‐natives may be embedded within remnant sites. Even though remnants sites would ideally be “pristine,” thorough sampling of the source population prerewilding is needed to assess the risks of spreading invasive species into areas in which they may not be present. The introduction of invasive species or pathogens is a risk for all reintroduction projects, regardless of whether they are single species or whole‐of‐community reintroductions. Rewilding should therefore only occur when natural recolonization seems to be impossible or exceedingly slow (Jourdan et al., 2019) (Figure 2). Although both single species and whole‐of‐community reintroductions carry inherent risks, the latter may provide benefits that outweigh negatives. For example, Haase and Pilotto (2019) argue that because single species freshwater macroinvertebrate reintroductions are only successful 62.5% of the time (Jourdan et al., 2019), whole community transfers may be the preferable method for stream restoration as they increase the likelihood of at least some species establishing.

Whole‐of‐community rewilding may also pose risks to the genetic integrity of species. For example, in a review of freshwater macroinvertebrate reintroduction projects, Jourdan et al. (2019) noted that the mixing of different evolutionary lines during reintroductions may diminish conservation goals. Flightless invertebrates (such as benthic freshwater macroinvertebrates and terrestrial mygalomorph spiders) are often dispersal‐constrained meaning genetic differentiation between populations is common. If a reintroduced population mixes with undetected individuals persisting in the restoration site, there could be hybridization between the two populations, which jeopardizes the integrity of the independent evolutionary lineages and their conservation. Jourdan et al. (2019) recommend genetic assessment of intraspecies diversity prereintroductions, and if this is not possible, choosing to reintroduce populations from the nearest remnant population, as we have previously recommended in this review.

## 9 BIOTIC BARRIERS NEED TO BE CONSIDERED

Invertebrate and microbial rewilding projects also need to consider the impact of biotic barriers and the challenges these barriers pose to successful establishment. For example, soil microbe rewilding projects have historically ignored the benefits of reinstating indigenous whole communities and instead focused on using single commercial species for restoration purposes (Asmelash et al., 2016). These commercial species are often non‐native and encounter competition with indigenous microbiota that have superior adaptions to local abiotic conditions. The potential impact of the biotic barrier is demonstrated by recent studies that compare singular non‐native AMF inoculation with indigenous whole‐of‐community rewilding. They found that introduced AMF were scarce as compared to indigenous AMF, indicating the former were ineffective at establishing and proliferating within the in situ soil community (Emam, 2016; Lance et al., 2019). This was expressed as increased soil function in revegetated areas inoculated with indigenous soil whole communities, resulting in greater soil Phosphorous concentration (Lance et al., 2019) and increased plant biomass (Emam, 2016). Conversely, the ecological consequences of commercial AMF outcompeting native species are unknown but could pose a threat for native soil biodiversity and ecosystem function (Hart et al., 2018).

Although there are limited examples of invertebrate rewilding, we can learn much from the success and failures of these projects. Reducing competition between in situ communities and rewilded communities by removing the former can have significant effects on the establishment of rewilded invertebrates and microbes. For example, a topsoil inoculation study looked at the difference in restoration success between areas where the topsoil and its resident soil community had been removed as compared to areas where the resident soil community was unaltered (Wubs et al., 2016). They found that when rewilded, whole soil communities were more likely to establish in areas where topsoil had been stripped and the competitive effects of resident soil communities removed, which manifested as a more successful restoration effort. Whether this is a general pattern, or dependent on the habitat in question, is not known. Benetková et al. (2020) speculated that it may be more appropriate to strip the resident community in forests as opposed to grasslands as soil formation is much faster under forests. They posit that other soil restoration projects (Moradi et al., 2018; van der Bij et al., 2018) were more successful than theirs due to their different rewilding methodology (Benetková et al. (2020) transplanted soil on top of the resident community as opposed to removing the resident community prior to the transplants). Further, literature on invertebrate translocations revealed that predation from species in the established community was a significant barrier to the establishment of reintroduced invertebrates (Bellis et al., 2019). This should also be a consideration for invertebrate and microbial rewilding.

## 10 UNEXPECTED CONSEQUENCES OF THE BIOTIC BARRIER

The biotic barrier can manifest unforeseen results during restoration. Remediation research has shown that the establishment of beneficial microbial inoculants in soil communities is not strictly correlated with measurable macro‐ecological outcomes (plant growth in this instance). At least two independent studies have reported beneficial plant growth outcomes even when the inoculant was lost from the soil (Kang et al., 2013; Liu et al., 2015). The beneficial effects are attributed to changes to the native community structure and function triggered by the addition, and subsequent demise, of the inoculum. This research highlights significant knowledge gaps in managing soil function and indicates that monitoring of soil communities postrewilding will be necessary to disentangle outcomes for ecosystem functions and the microbial community. Addressing these knowledge gaps can include more specific instances of rewilding soil microbial communities that extend beyond current soil inoculation methodologies (Box 2).

BOX 2 **Rewilding microbes to boost carbon cycling**

Soil microbes are key drivers of the global cycling of Soil Organic Carbon (SOC). They both deplete SOC through respiration and accumulate SOC through growth and by stabilizing soil aggregates (Anthony et al., 2020). Ecto‐ and endo‐mycorrhizal fungi are particularly important for the accumulation of SOC as their hyphae stabilize soil aggregates, making SOC inaccessible for other microbes and limiting respiration (Wei et al., 2019). Restoration of the natural carbon cycle in revegetated areas is therefore highly dependent on the return of soil microbiota. Although some soil communities in restoration areas track toward remnant sites (Barber et al., 2017), soil microbes differ in their dispersal capabilities according to the presence or absence of traits needed to survive during airborne dispersal such as spore formation and pigment production (Choudoir et al., 2018). Consequently, dispersal distances vary greatly between species, with some fungi exhibiting effective dispersal ranges of only ~1 km (Peay et al., 2010).

Like invertebrates, the return of soil microbes and the functions they provide to revegetated “habitat islands” on degraded farmlands is often assumed to occur passively (Box 1). However, restoration projects may benefit from actively rewilding soil microbes. This could both overcome dispersal constraints and tailor the reconstructed microbe community to a desired trajectory. Local paddock trees are often the last remaining remnant trees on degraded farms. They are potential reservoirs of soil carbon cycling taxa as they contain mycorrhizal fungi and bacterial species adapted to competitive dynamics within local conditions (Wood et al., 2018). This provides a competitive advantage over communities already established in revegetated areas and increases the likelihood of rewilded communities overcoming the biotic barrier.

**FIGURE 4:**
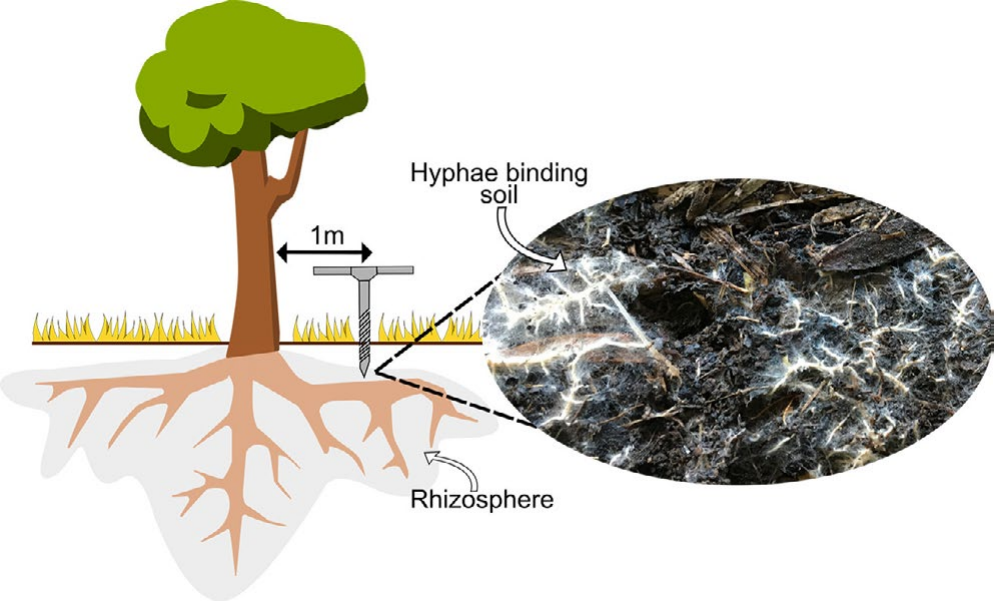
Collecting soil from the whole rhizosphere region of large established trees is impractical. Collecting rhizosphere communities by sampling 1 m out from the base of trees using a soil corer is a viable methodologic approach and is a more targeted way of rewilding microbial communities than current soil inoculation studies. Previous research has demonstrated that rhizosphere signatures can be detected using this approach for microbe communities from rainforest plant species despite the complex overlapping root networks (Wood et al., 2020)

Rewilding soil microbial communities would entail moving soil from the rhizosphere (area of soil in contact with roots) of a local paddock tree and scattering this around the base of revegetated plants (Figure 4). Local paddock trees may be particularly useful in highly modified systems where remnant patches are nonexistent and could contain the last locally adapted source of remnant microbial communities.

## 11 HOW CAN WHOLE‐OF‐COMMUNITY REWILDING AID CONSERVATION?

Although the emphasis of whole‐of‐community rewilding often falls on reinstating function (Table 1), it may play a valuable role in future conservation efforts. For example, the alarming rate at which invertebrates are declining worldwide has only recently received public attention (Eisenhauer, 2019), with some estimating populations of terrestrial invertebrates are declining roughly 9% per decade (van Klink et al., 2020). Although there are risks associated with whole‐of‐community rewilding, this underutilized method for restoring communities can provide a way to rapidly improve biodiversity, bypassing the slow method of species‐by‐species reintroductions found in current rewilding projects. Evidence for this can be found in the most common form of whole‐of‐community rewilding, topsoil inoculation studies. Transplants of whole soil communities can improve the biodiversity and density of mites and springtails (van der Bij et al., 2018; Wubs et al., 2016), the abundance of wetland macroinvertebrates (Brown et al., 1997), soil nematode abundance (Benetková et al., 2020), and soil macrofauna abundance (Moradi et al., 2018). Similarly, Haase and Pilotto (2019) noted that their method of rewilding whole communities of stream invertebrates introduced 45 taxa from remnant streams that were absent in partially restored recipient streams.

## 12 CONCLUSION: TOWARD GREATER USE OF INVERTEBRATES AND MICROBES

If ecological restoration is to move forward as a more complete science, it is critical that we further investigate the role that rewilded invertebrates and microbes play during restoration. Recent advances in the field of restoration ecology have explored both the potential of whole‐of‐community rewilding as a restorative tool (Wubs et al., 2016) and how benthic stream invertebrates can be translocated as whole communities (Dumeier et al., 2020). We hope that the ideas and practical case studies proposed in this article will spur further empirical testing of whole‐of‐community rewilding which extend beyond soil inoculation studies. Monitoring throughout the life of invertebrate and microbial rewilding projects will be vital to determining their efficacy and the conditions under which it will enhance recovery rates. Ecosystem functions mediated by rewilded vertebrates can vary across abiotic gradients (Decker et al., 2019). Whether the restorative potential of rewilded invertebrates and microbes varies spatially, and temporally, should therefore be explored.

Restoration projects currently overlook two groups that make up the bulk of biodiversity (Kollmann et al., 2016). This brings into question whether most restoration projects are failing to attain their end goal: the reinstatement of biodiversity and ecosystem function in its entirety. Our argument for rewilding invertebrates and microbes addresses this shortfall, but we stress that their use should be considered for the novel advantages alone. Whole‐of‐community rewilding is a unique and potentially very powerful tool that land managers are largely unaware of. A greater incorporation of invertebrates and microbes in rewilding projects may also simultaneously answer the resounding call for more thorough monitoring of this underappreciated group (Eisenhauer, 2019). This would also help to fill substantial gaps in baseline knowledge of what species are present and what their functional role is before they are lost, thus assisting future recovery efforts of globally declining invertebrate populations (Klink et al., 2020). We hope that the ideas raised in this discussion engender a greater appreciation for the restoration and rewilding potential that invertebrates and microbes deserve. This can help mold restoration ecology into a more holistic science that values the role of all biota, irrespective of size.

## CONFLICTS OF INTEREST

None declared.

## AUTHOR CONTRIBUTIONS

**Peter Contos:** Conceptualization (lead); writing–original draft (lead); writing–review and editing (lead). **Jennifer L. Wood:** Conceptualization (supporting); writing–original draft (lead); writing–review and editing (supporting). **Nicholas P. Murphy:** Conceptualization (supporting); writing–review and editing (supporting). **Heloise Gibb:** Conceptualization (lead); writing–original draft (lead); writing–review and editing (lead).

## Data Availability

No data were used.
